# CD40L and IL-4 suppress NK cell-mediated antibody-dependent cellular cytotoxicity through the HLA-E:NKG2A axis

**DOI:** 10.1093/immadv/ltaf029

**Published:** 2025-08-27

**Authors:** Lara V Graham, Ludmila Horehajova, Marco V Haselager, Jack G Fisher, Jamie Lee Roos, Russell B Foxall, Mel John, Kerry L Cox, Robert J Oldham, Martin C Taylor, Margaret Ashton-Key, Ben Sale, Laura G Bartlett, Ali Roghanian, Eric Eldering, Andres F Vallejo, Francesco Forconi, Salim I Khakoo, Mark S Cragg, Matthew D Blunt

**Affiliations:** School of Clinical and Experimental Sciences, University of Southampton, Southampton, United Kingdom; School of Clinical and Experimental Sciences, University of Southampton, Southampton, United Kingdom; Department of Experimental Immunology, Amsterdam UMC, University of Amsterdam, Meibergdreef, The Netherlands; Lymphoma and Myeloma Centre Amsterdam, LYMMCARE, Amsterdam, The Netherlands; Cancer Centre Amsterdam, Amsterdam Institute for Infection and Immunity, Amsterdam, The Netherlands; Amsterdam Institute for Infection and Immunity, Cancer Immunology, Amsterdam, The Netherlands; School of Clinical and Experimental Sciences, University of Southampton, Southampton, United Kingdom; Department of Experimental Immunology, Amsterdam UMC, University of Amsterdam, Meibergdreef, The Netherlands; Lymphoma and Myeloma Centre Amsterdam, LYMMCARE, Amsterdam, The Netherlands; Cancer Centre Amsterdam, Amsterdam Institute for Infection and Immunity, Amsterdam, The Netherlands; Amsterdam Institute for Infection and Immunity, Cancer Immunology, Amsterdam, The Netherlands; School of Cancer Sciences, University of Southampton, Southampton, United Kingdom; Antibody and Vaccine Group, Centre for Cancer Immunology, Faculty of Medicine, University of Southampton, Southampton, United Kingdom; School of Clinical and Experimental Sciences, University of Southampton, Southampton, United Kingdom; School of Cancer Sciences, University of Southampton, Southampton, United Kingdom; Antibody and Vaccine Group, Centre for Cancer Immunology, Faculty of Medicine, University of Southampton, Southampton, United Kingdom; School of Cancer Sciences, University of Southampton, Southampton, United Kingdom; Antibody and Vaccine Group, Centre for Cancer Immunology, Faculty of Medicine, University of Southampton, Southampton, United Kingdom; School of Cancer Sciences, University of Southampton, Southampton, United Kingdom; Antibody and Vaccine Group, Centre for Cancer Immunology, Faculty of Medicine, University of Southampton, Southampton, United Kingdom; School of Cancer Sciences, University of Southampton, Southampton, United Kingdom; Antibody and Vaccine Group, Centre for Cancer Immunology, Faculty of Medicine, University of Southampton, Southampton, United Kingdom; School of Cancer Sciences, University of Southampton, Southampton, United Kingdom; School of Clinical and Experimental Sciences, University of Southampton, Southampton, United Kingdom; School of Cancer Sciences, University of Southampton, Southampton, United Kingdom; Antibody and Vaccine Group, Centre for Cancer Immunology, Faculty of Medicine, University of Southampton, Southampton, United Kingdom; Department of Experimental Immunology, Amsterdam UMC, University of Amsterdam, Meibergdreef, The Netherlands; Lymphoma and Myeloma Centre Amsterdam, LYMMCARE, Amsterdam, The Netherlands; Cancer Centre Amsterdam, Amsterdam Institute for Infection and Immunity, Amsterdam, The Netherlands; Amsterdam Institute for Infection and Immunity, Cancer Immunology, Amsterdam, The Netherlands; School of Clinical and Experimental Sciences, University of Southampton, Southampton, United Kingdom; School of Cancer Sciences, University of Southampton, Southampton, United Kingdom; Haematology Department, Cancer Care Directorate, University Hospital Southampton NHS Trust, Southampton, United Kingdom; School of Clinical and Experimental Sciences, University of Southampton, Southampton, United Kingdom; School of Cancer Sciences, University of Southampton, Southampton, United Kingdom; Antibody and Vaccine Group, Centre for Cancer Immunology, Faculty of Medicine, University of Southampton, Southampton, United Kingdom; School of Clinical and Experimental Sciences, University of Southampton, Southampton, United Kingdom

**Keywords:** NK cells, lymph nodes, ADCC, rituximab, NKG2A, HLA-E, monalizumab

## Abstract

**Background:**

Anti-CD20 antibodies are first-line treatments for B cell malignancies. Natural killer (NK) cells are important mediators of anti-CD20 antibody efficacy in humans through antibody-dependent cellular cytotoxicity (ADCC). In B cell malignancies, the lymph nodes are a critical site of pathology and the T cell-derived signals CD40L and IL-4 within the lymph node microenvironment can mediate tumour proliferation, survival and resistance to pro-apoptotic therapy. CD40L and IL-4 have recently been shown to inhibit NK cell activation against chronic lymphocytic leukaemia (CLL) cells via the HLA-E:NKG2A immune checkpoint axis. However, the effect of these signals on NK cell-mediated ADCC of malignant B cells is unclear.

**Methods:**

Using a combination of clinical samples, murine models, flow cytometry, immunoblotting, immunohistochemistry, ELISA, bioinformatics and functional assays, we examined the impact of lymph node-mimicking conditions on NK cell-mediated ADCC against malignant B cells. Exogenous CD40L and IL-4 were used to mimic T-B cell interactions in 2D malignant B cell cultures, in addition to a 3D spheroid model of T cell-dependent CLL proliferation.

**Results:**

CD40L and IL-4 increased HLA-E expression on the surface of primary CLL cells and non-Hodgkin’s lymphoma (NHL) cell lines, and this decreased NK cell-mediated ADCC via ligation of the inhibitory receptor NKG2A. High HLA-E surface expression was observed in lymph node FFPE sections of CLL and NHL patients and in a 3D *ex vivo* lymph node-mimicking model of CLL. NKG2A blockade potentiated NK cell-mediated ADCC against malignant B cells treated with CD40L and IL-4 and improved anti-CD20 antibody therapy in a murine model of B cell lymphoma.

**Conclusion:**

These results reveal a novel mechanism of resistance to anti-CD20 therapy in B cell malignancies and demonstrate that the combination of anti-NKG2A with anti-CD20 could improve the treatment of patients with CLL or NHL.

## Graphical Abstract

**Graphical Abstract:**
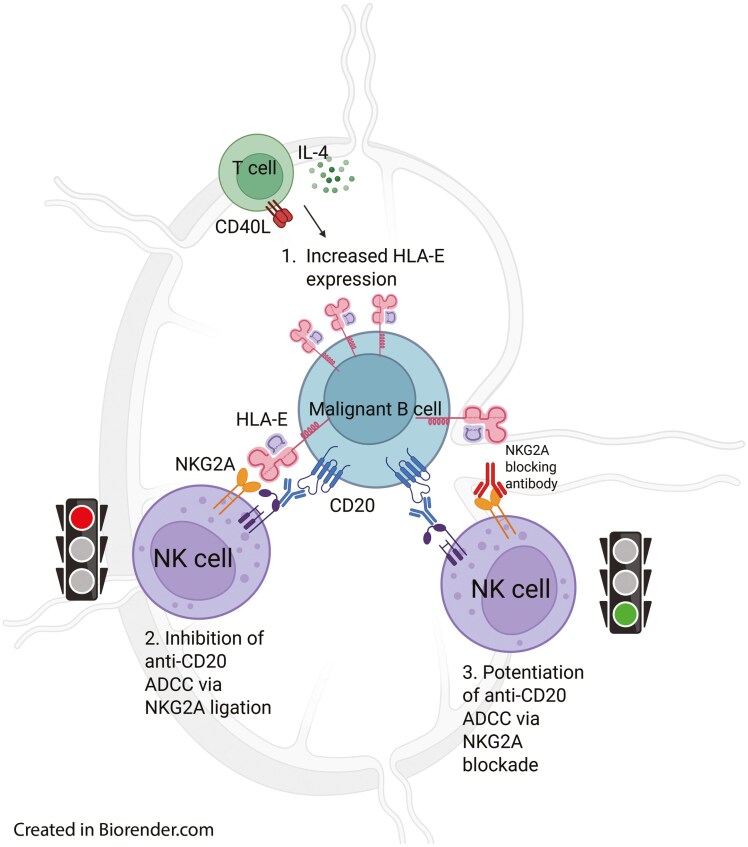

## Introduction

The anti-CD20 monoclonal antibodies (mAb) rituximab and obinutuzumab have revolutionized the treatment of B cell malignancies and form part of first-line therapies for NHL and chronic lymphocytic leukaemia (CLL). Rituximab is a chimeric mAb with a human IgG1 Fc domain [1]. Engagement of the Fc portion with Fc gamma receptors (FcγR) expressed on monocytes/macrophages and NK cells induces antibody-dependent cellular phagocytosis and antibody-dependent cellular cytotoxicity (ADCC) against CD20-expressing target cells. Obinutuzumab is a humanized IgG1 mAb engineered to induce more potent effector cell activation via an afucosylated Fc region that has enhanced affinity for FcγIII (CD16a/b) [2].

NK cells express CD16a and are considered the predominant inducers of ADCC in humans. NK cells are lymphocytes of the innate immune system and function to directly lyse tumour cells through the release of cytotoxic granules and ligation of death receptors, as well as modulation of other immune cells via release of chemokines and cytokines [3]. Due to these potent effector mechanisms and the ability to be activated via IgG-mediated CD16a ligation, NK cells are thought to contribute to the efficacy of anti-CD20 mAb immunotherapy in patients. For example, higher NK cell counts in the peripheral blood of diffuse large B cell lymphoma (DLBCL) and follicular lymphoma (FL) patients have been shown to correlate with better prognosis with rituximab plus chemotherapy (R-CHOP) treatment [4, 5]. In addition, higher expression of NK cell-associated gene signatures in tumours of lymphoma patients prior to treatment correlated with improved progression-free survival [5]. More recently, adoptive transfer strategies using *ex vivo* expanded NK cells from healthy donors combined with rituximab have been investigated in a clinical trial for the treatment of B cell malignancies [6]. Despite the success of anti-CD20 therapies, mechanisms of resistance can arise that limit antibody-dependent effector mechanisms and, ultimately, efficacy in patients [7, 8].

Both NHL and CLL can arise from malignantly transformed B cells in the lymph nodes [9, 10]. Moreover, the lymph nodes represent a critical pro-survival niche for these diseases [11, 12]. Key pathogenic factors are the T follicular helper cell (T_FH_)-derived signals CD40 ligand (CD40L) and IL-4, which have been shown to promote survival of NHL cells [13, 14] and primary CLL cells *ex vivo* [15]. Moreover, these signals induce resistance of malignant B cells to cytotoxic agents [16–18], signalling inhibitors (ibrutinib), and BH3 mimetics (venetoclax) [19–21]. This means the lymph nodes can be a source of minimal residual disease in patients with B cell malignancies, which increases the risk of relapse [22]. It is therefore critical to understand tumour cell depletion within the lymph node microenvironment in order to develop novel treatment strategies to overcome these resistance mechanisms and improve patient outcomes.

We have recently shown that CD40L and IL-4 can inhibit NK cell activation against primary CLL cells via upregulation of HLA-E, the ligand for the NK cell inhibitory receptor NKG2A [23]. When bound to its ligand HLA-E, NKG2A inhibits NK cell activating signalling via recruitment of SHP-1 tyrosine phosphatase [24]. However, it is unknown how this may affect antibody therapy for CLL and NHL. In this study, we therefore investigated the effect of CD40L and IL-4 on ADCC evoked by rituximab and obinutuzumab and determined the impact of modulating the HLA-E:NKG2A axis under these conditions.

## Materials and methods

### Primary PBMC samples and cell culture

Peripheral blood mononuclear cells (PBMCs) were obtained from CLL patients (Supplementary Table S1) recruited in the ‘real world’ observational study at the University of Southampton (NIHR/UKCRN ID: 31076, CI F.Forconi) or Departments of Hematology and Pathology of the Academic Medical Center (AMC). Written informed consent was obtained in accordance with ethics committee approvals under the Declaration of Helsinki or the AMC Ethical Review Biobank Board under the number METC 2013/159. Diagnosis of CLL was according to the 2008 International Workshop on CLL (IWCLL2008)/National Cancer Institute criteria. Diagnosis was confirmed by a flow cytometry ‘Matutes score’ >3 in all cases [25]. Phenotypic and immunogenetic characteristics (tumour Immunoglobulin heavy chain (IGHV) usage and mutational status) were determined as previously described [26]. Healthy donor PBMCs were obtained from volunteers with ethical approval from the National Research Ethics Committee (REC reference 19/WM/0262 or 24/PR/0245). PBMCs from healthy donors were cultured in RPMI 1640 (Gibco) supplemented with 1% penicillin-streptomycin (Sigma) and 10% fetal bovine serum (FBS, Sigma) (R10) with 10 ng/mL IL-15 (R&D systems) overnight. NK cells were isolated from healthy donor PBMCs using the Miltenyi Biotech human NK cell isolation kit and were cultured in Iscove’s Modified Dulbecco’s Medium supplemented with 1% penicillin-streptomycin and 10% FBS (I10) with 10 ng/mL IL-15 overnight, or human NK MACS medium supplemented with 5% human male AB serum (Merck), 1% MACS supplement and 500 IU/ml human IL-2 (all Miltenyi Biotech) for 14–21 days prior to assays. Cell lines (Ramos, Raji, SU-DHL-6, SU-DHL-4, 721.174) were maintained in R10 and tested at regular intervals for mycoplasma. JeKo-1 cells were maintained in RPMI 1640 supplemented with 1% penicillin-streptomycin and 20% FBS.

### Flow cytometry

FACS buffer (PBS, 1% bovine serum albumin, 0.05% sodium azide) with 10% male AB human serum (Sigma) or mouse TruStain FcX (Biolegend) was used to block Fc receptors of human or murine cells, respectively, for 15 min. Cells were then stained for 30 min at 4°C with antibodies outlined in Supplementary Table S2. Data was collected on a BD FACS Aria II using FACSDiva software or BD Accuri C6 and analysed with FlowJo v10.8.1 (all BD Biosciences).

### Assessment of NK cell function

#### NK cell-mediated target cell lysis

Target cells were stained with Cell Trace Violet or Far-Red Cell Proliferation Kit (Invitrogen^TM^) before treatment with 300 ng/ml CD40L and 10 ng/ml IL-4 (both R&D Systems) for 24 h. In indicated experiments, 500 nM selinexor (Karyopharm Therapeutics) or DMSO control was also added to cells 30 min after CD40L and IL-4. To minimize differences in target cell viability, target cells were treated with 30 μM caspase inhibitor Q-VD-OPh (Sigma) 1 h after the addition of CD40L and IL-4. For ADCC experiments, target cells were incubated with rituximab (hIgG1, in-house, or Roche), obinutuzumab (hIgG1, Southampton General Hospital Pharmacy or Leinco Technologies) or isotype control (AT171-2, hIgG1, in-house) for 20 min at 37°C. During NKG2A blockade experiments, NK cells from healthy donors were incubated with 10 μg/ml anti-NKG2A clone Z199 (mIgG2b, Beckman-Coulter), 15 μg/ml anti-NKG2A monalizumab (hIgG4, Selleckchemicals) or their respective isotype controls (mIgG2b, Biolegend, or hIgG4, in-house) for 20 min at room temperature. NK cells were then co-cultured with target cells at the indicated effector:target (E:T) ratios for 4–48 h at 37°C. For 3D experiments, cells were co-cultured in ultra-low-attachment (ULA) plates (Corning) and were centrifuged at 200 × *g* for 10 min prior to incubation. Cells were stained with propidium iodide (Invitrogen^TM^) or DioC6 and TO-PRO-3 (both ThermoFisher Scientific) to assess lysis of the cells by flow cytometry. Specific lysis was calculated using the formula:


$$\begin{array}{lll} & Specif\;\;ic\;lysis=\; & \;\frac{\left(\%\;target\;cell\;lysis\;in\;coculture\right)-\left(\%\;spontenous\;target\;cell\;lysis\right)}{100-\left(\%\;spontaneous\;target\;cell\;lysis\right)} \end{array}$$

#### NK cell degranulation

Target cells (Ramos and primary CLL) were treated with CD40L and IL-4 as above. To assess ADCC, CLL samples were incubated with 10 μg/ml rituximab for 20 min at 37°C and washed twice in R10 medium. Healthy donor PBMCs were incubated with 10 ng/mL IL-15 (R&D Systems) overnight and stained with 0.17 μg/ml α-CD107a-eFluor660 (eBioH4A3, Invitrogen) for 10 min at room temperature. Healthy donor PBMCs were then added to the target cells at a 5:1 E:T for 4 h at 37°C, with the addition of Golgistop (BD Biosciences) after 1 h of co-culture. Data was collected using flow cytometry as above. Data presented represent CD107a (%) values minus the no target control.

#### IFNγ ELISA

Supernatants from isolated NK cell co-culture with Raji cells were collected after 24 h. IFNγ secretion (pg/mL) was assessed using the human IFNγ uncoated ELISA kit (Invitrogen) in accordance with the manufacturer’s instructions. Plates were scanned at 450 nm using a Multiscan FC (Thermo Scientific).

### Long-term 3D spheroid culture

CLL samples were cultured in I10 in ULA plates (Corning) with 25 ng/mL IL-2, IL-15 (both peprotech) and IL-21 (Gibco) with 1 μg/ml CpG (Invivogen) as previously described [27]. In the indicated experiments, 5 μg/ml anti-CD40 antagonist (clone 341G2, hIgG4, in-house) was added to the culture. Plates were centrifuged for 10 min at 200xg and incubated at 37°C and 5% CO_2_ to allow spheroid formation. In the indicated experiments, CLL cells were isolated using the Miltenyi Biotech human B-CLL cell isolation kit before subsequent culture as above.

### Murine B cell lymphoma tumour immunotherapy

About 8–12-week-old female BALB/c mice, originally obtained from Charles River, were maintained in the local animal research facility. Animal experiments were approved by the University of Southampton Ethical Committee and performed in accordance with UK Home Office license guidelines under PIL (I27545999) and PPL (PP5396109).

BCL_1_ murine B cell lymphoma cells were maintained by passage in BALB/c mice. BCL_1_ tumour cells were obtained from freshly harvested spleens, and 10^4^ BCL_1_ cells were injected intravenously (i.v.) into the tail veins of BALB/c mice. Mice were then injected with anti-NKG2A (clone 20D5, rIgG2a, 200 μg, i.v., Assay Genie) on days 6 and 13 and anti-CD20 (clone 18B12, mIgG2a, 200 μg, intraperitoneal (i.p.), produced in-house) on day 7 or respective isotype controls (produced in-house). Blood was collected at the indicated timepoints via tail lancing. Tumour growth was monitored by palpation of the spleen over time for mice and humane endpoint was spleen palpation grade 3.

### Immunoblotting

After treatment with CD40L and IL-4 for 24 h, CLL cells and cell lines were assessed by immunoblotting as previously described [28] using antibodies against HLA-E (polyclonal Rabbit IgG, Sigma), MCL-1 (D35A5, rabbit IgG, Cell Signalling Technology) or β-actin (8H10D10, mIgG2b, Cell Signalling Technology). Bands were visualized using Pierce^TM^ ECL Western Blotting substrate (Thermo Scientific) and imaged with the ChemiDoc-It imaging system (UVP).

### Immunohistochemistry

Formalin-fixed, paraffin-embedded (FFPE) sections (4 μm) of consented human CLL and NHL patient lymph nodes pretreatment were provided by Cellular Pathology, University Hospital Southampton NHS Foundation Trust (Supplementary Table S3). Ethical approval was obtained by Southampton University Hospitals NHS Trust from Southampton and Southwest Hampshire Research Ethics Committee (REC reference 23/NW/0060). Sections were stained using the fully automated BOND-RX immunohistochemistry staining instrument (Leica Microsystems), in accordance with the manufacturer’s instructions. In brief, sections were deparaffinized, pretreated for heat-induced antigen retrieval and stained for HLA-E (MEM-E/02, Abcam) or CD20 (L26, DAKO) in BOND Primary Antibody Diluent (Leica Microsystems). The antibody was then bound to the polyHRP IgG reagent before incubation with 3,3′-diaminobenzidine. Slides were digitalized using the Zeiss Axioscan 7 and images were taken using Zenv3.10 Lite (Zeiss).

### Statistics

Statistical analysis was performed using GraphPad Prism software (version 8.0) or Python v3.11. Paired Student’s two-tailed *t*-test or multiple T tests were used to compare data with two groups and one-way or two-way ANOVA was used to compare data with more than 2 groups. The log-rank Mantel-Cox test was used to analyse differences in survival. Data was considered statistically significant at *P* < .05.

### Bioinformatics

Bioinformatic analysis is described in the Supplementary information.

## Results

### The lymph node-associated signals CD40L and IL-4 suppress NK cell-specific lysis against primary CLL cells and NHL cell lines

**Figure 1.**
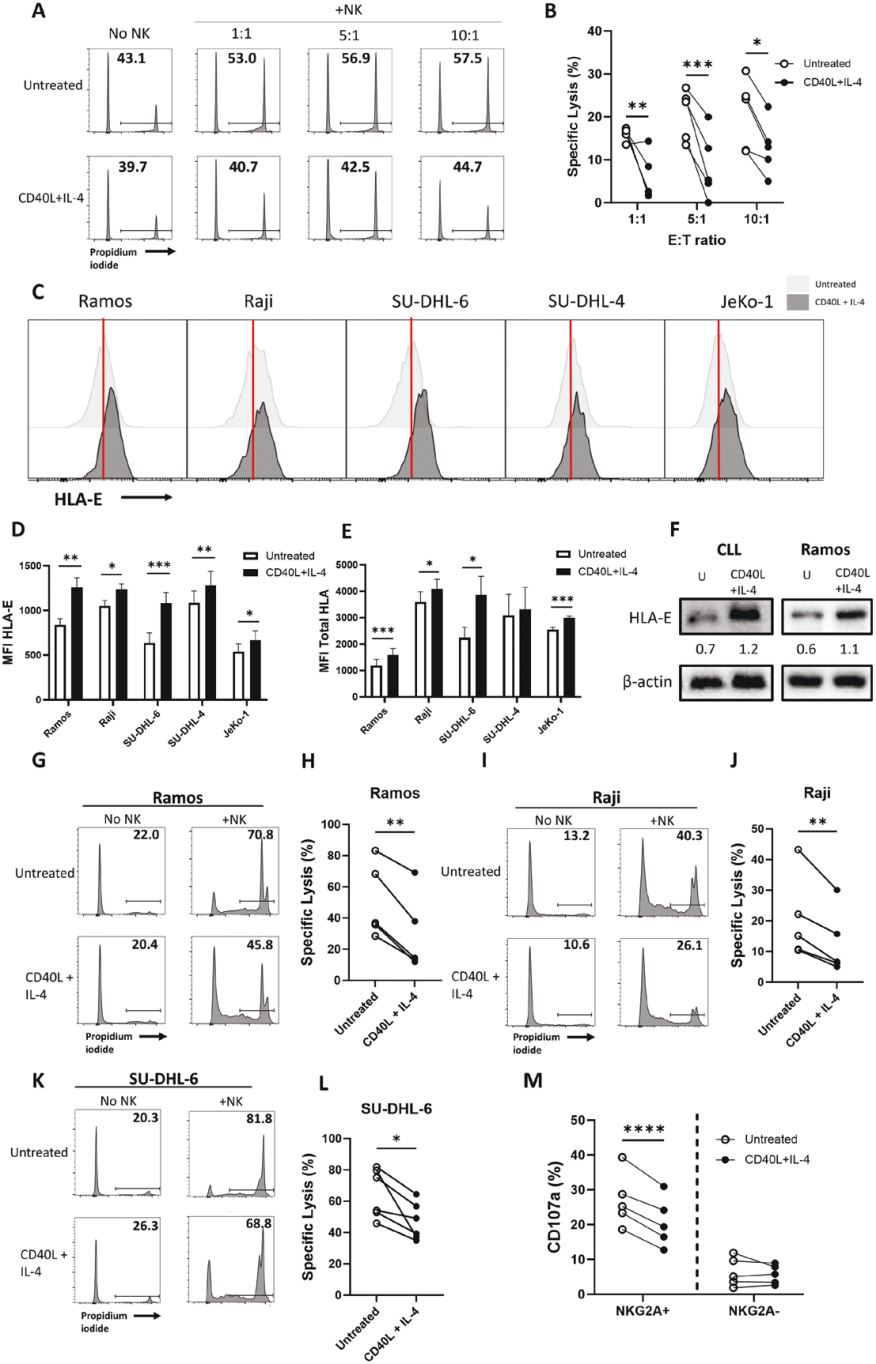
CD40L and IL-4 suppress NK cell-mediated lysis of B cell leukaemia and lymphoma cells. (a and b) Primary CLL cells (>90% tumour) were treated with 300 ng/ml CD40L and 10 ng/ml IL-4 or left untreated for 24 h at 37°C before co-culture with NK cells from healthy donors for a further 6 h at 1:1, 5:1, and 10:1 effector:target (E:T) ratios. Propidium iodide was used to measure the proportion of lysed CLL cells by flow cytometry. Representative FACS plots of raw lysis data are shown in (a) and summarized data of NK cell-specific lysis are shown in (b) (*n* = 5). (c–f) NHL cell lines Ramos, Raji, SU-DHL-6, SU-DHL-4, and JeKo-1 were treated with 300 ng/ml CD40L and 10 ng/ml IL-4 for 24 h. Representative FACS plots of HLA-E expression shown in (c) and data is represented as mean±SEM for HLA-E (d) and total HLA (e) (*n* = 6). (f) Abundance of HLA-E protein in primary CLL and Ramos cells as measured by immunoblotting (representative of three repeats). (g–l) Ramos, Raji, and SU-DHL-6 cells were treated as above before co-culture with NK cells from healthy donors at 1:1 E:T ratio for 24 h. Propidium iodide was used to measure the proportion of lysed NHL cells. Representative FACS plots of raw lysis data shown in (g), (i), and (k) and summarized data of NK cell-specific lysis shown in (h), (j), and (l) (*n* = 5–6). (m) Ramos cells were treated as above before co-culture with healthy donor PBMCs at a 5:1 E:T for 4 h. Degranulation (CD107a expression) of NKG2A+ and NKG2A− NK cells was assessed by flow cytometry and data are shown as CD107a expression (%) minus the no target control. Statistical significance was calculated by two-way ANOVA with Sidak’s correction for multiple comparisons or multiple paired T tests with the Holm-Sidak’s multiple comparisons correction. *P < .05, **P < .01, ***P < .001, ****P < .0001.

We previously showed that CD40L and IL-4 increased HLA-E expression on the surface of primary CLL cells and inhibited NKG2A+ NK cell degranulation and IFNγ expression [23]. However, it was unknown whether this also impacted the ability of NK cells to lyse CLL cells. CD40L and IL-4 resulted in less death of CLL cells in the absence of NK cells (Fig. 1a), consistent with previous reports [20], and significantly reduced NK cell specific lysis of primary CLL cells at all E:T ratios tested (1:1 *P* < .01, 5:1 *P* < .001, 10:1 *P* < .05) (Fig. 1a and b). As NHLs also have significant lymph node involvement, we also tested the effect of CD40L and IL-4 on NHL cell lines. Consistent with previous results in primary CLL [23], HLA-E expression also increased on the surface of NHL cell lines in response to CD40L and IL-4 (Fig. 1c and d). Total HLA also increased on the surface of Ramos (*P* < .001), Raji (*P* < .05), SU-DHL-6 (*P* < .05) and JeKo-1 (*P* < .001) cell lines (Fig. 1e). In addition, we confirmed increased HLA-E protein abundance following CD40L and IL-4 treatment of primary CLL and Ramos cells by immunoblotting (Fig. 1f). CD40L and IL-4 significantly reduced specific lysis of NHL cell lines (Raji *P* < .01, Ramos *P* < .01, SU-DHL-6 *P* < .05) (Fig. 1g–l). To confirm the relationship between the effect of CD40L and IL-4 on NK cell response and HLA-E, we assessed the effect of these signals on degranulation (CD107a expression) of NKG2A+ versus NKG2A- NK cells (Supplementary Fig. S1). NKG2A- NK cells showed lower activation against Ramos cells overall compared to NKG2A+ NK cells (Fig. 1m), which may reflect differences in NK cell education mediated by NKG2A [29]. CD40L and IL-4 reduced degranulation of NKG2A+ NK cells against Ramos cells (*P* < .0001), but there was no significant effect on the NKG2A− population (Fig. 1m). Overall, these data strongly suggest that CD40L and IL-4 inhibit NK cell-mediated lysis of primary CLL cells and NHL cell lines via the HLA-E:NKG2A axis.

### CD40L and IL-4 suppress NK cell-mediated ADCC against primary CLL and NHL cell lines

**Figure 2.**
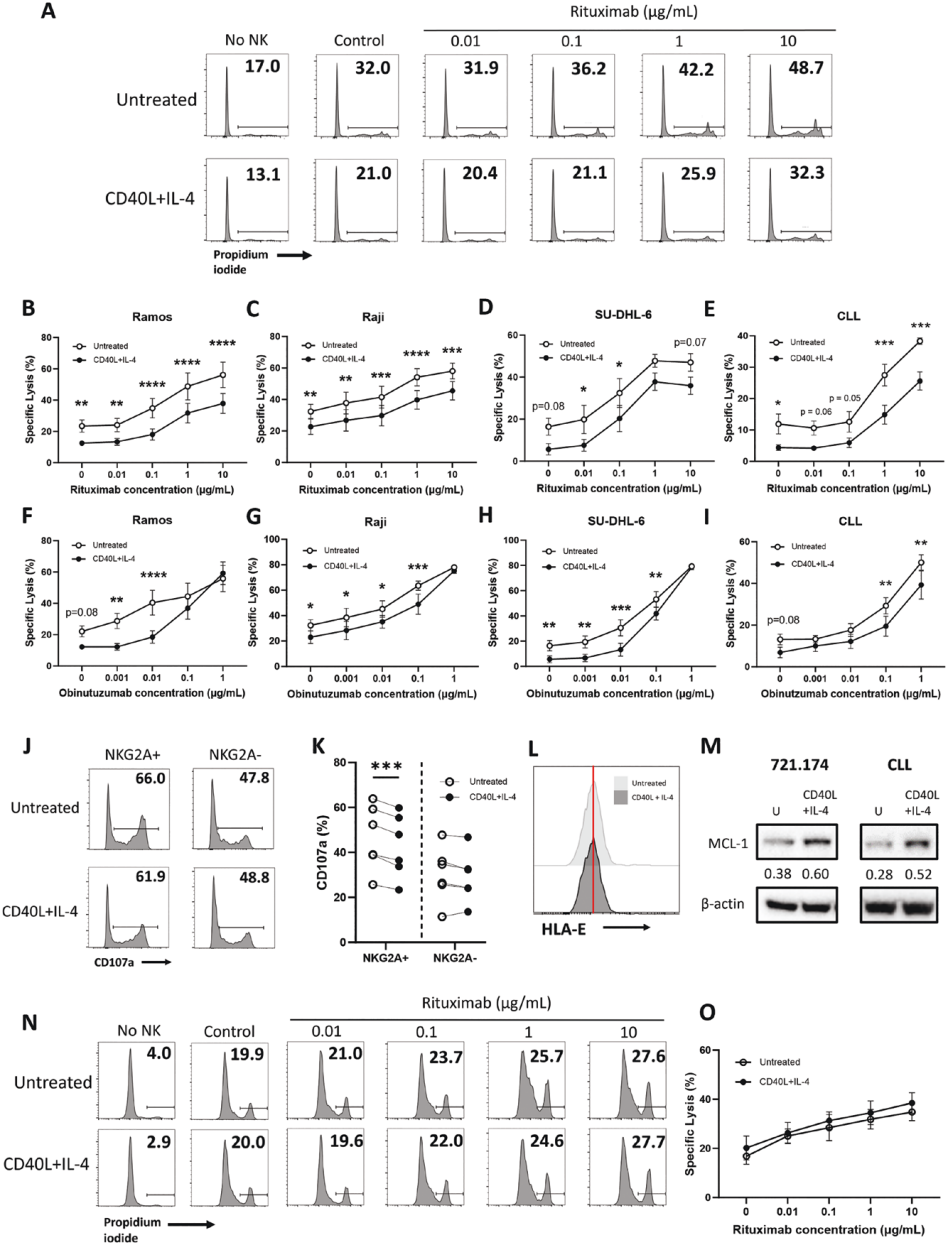
CD40L and IL-4 suppress NK cell-mediated ADCC of leukaemia and lymphoma cells. (a–i) Target cells were treated with 300 ng/ml CD40L and 10 ng/ml IL-4 or left untreated for 24 h before incubation with rituximab or obinutuzumab at the indicated concentrations or isotype control (10 or 1 μg/ml) and co-culture with NK cells from healthy donors at a 1:1 (Raji, CLL) or 1:5 (Ramos, SU-DHL-6) E:T ratio. Representative FACS plots of raw lysis data of Ramos cells are shown in (a) and NK cell-specific lysis is summarized as mean±SEM in the presence of increasing concentrations of rituximab (b–e) and obinutuzumab (f–i) (*n* = 3–5). (j and k) Primary CLL cells (>90% tumour) were treated with CD40L and IL-4 as above before co-culture with healthy donor PBMCs at a 5:1 E:T for 4 h in the presence of rituximab (10 μg/ml). Degranulation of NKG2A+ and NKG2A− NK cells was assessed by flow cytometry (*n* = 6). (l) 721.174 cells were treated with CD40L and IL-4 as above and HLA-E expression was assessed by flow cytometry. (m) 721.174 and primary CLL cells were treated with CD40L and IL-4 as above and MCL-1 protein abundance was assessed by immunoblotting (values under blot represent densitometry). Data for primary CLL are shown as a positive control. Representative of four repeats for 721.174 cells and three different donors for CLL. (n and o) 721.174 cells were treated with CD40L and IL-4 as above before incubation with rituximab at the indicated concentrations and co-culture with NK cells from healthy donors at a 1:10 E:T for 4 h. Representative FACS plots shown in N and NK cell specific lysis is summarized as mean±SEM in the presence of increasing concentrations of rituximab shown in (o) (*n* = 5). Statistical significance was calculated using two-way ANOVA with Sidak’s correction for multiple comparisons. *P < .05, **P < .01, ***P < .001, ****P < .0001.

Because NK cells contribute to the efficacy of anti-CD20 mAb therapies in B cell malignancies, we sought to investigate whether CD40L and IL-4 would also impact NK cell-mediated ADCC in combination with rituximab or obinutuzumab, which are in clinical use. In the presence of multiple concentrations of rituximab, CD40L, and IL-4 significantly decreased specific lysis against Ramos (Fig. 2a and b), Raji (Fig. 2c) and SU-DHL-6 (Fig. 2d) NHL cell lines, as well as against primary CLL (Fig. 2e). Similar effects were observed in the presence of obinutuzumab (Fig. 2f–i); however, the inhibitory effect was lost at the highest concentration of mAb, indicating inhibition of NK cell effector function by CD40L and IL-4 may be overcome by sufficient activation of NK cells. Inhibition of NK cell-mediated ADCC with rituximab against CLL was observed in the NKG2A+ population (*P* < .001) and not the NKG2A− population (Fig. 2j and k), again indicating that the observed inhibition is through the HLA-E:NKG2A axis. To confirm the importance of surface HLA-E, we utilized the TAP-deficient lymphoblastoid cell line 721.174 that has very low HLA-E surface expression due to impaired antigen processing machinery [30]. CD40L and IL-4 had no effect on surface expression of HLA-E on 721.174 cells (Fig. 2l) although increased expression of MCL-1 protein was evident (Fig. 2m), indicating that these cells were responsive to CD40L and IL-4. CD40L and IL-4 treatment had no effect on NK cell-mediated ADCC against 721.174 cells (Fig. 2n and o). This data indicates that the inhibitory effects of CD40L and IL-4 on ADCC are mediated by upregulation of surface HLA-E expression on target cells and not by upregulation of antiapoptotic proteins.

### HLA-E is highly expressed in the lymph nodes of patients with CLL and NHL and is upregulated by microenvironmental signals in a patient-derived 3D spheroid model

**Figure 3.**
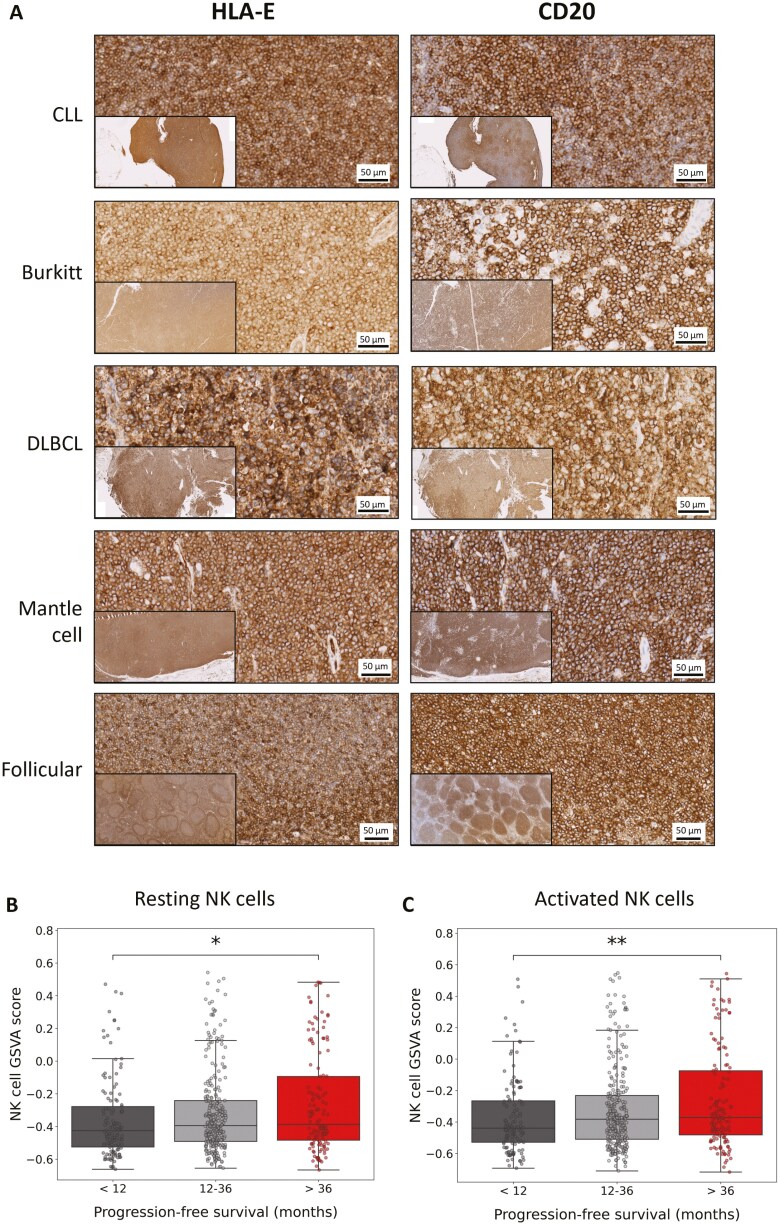
Identification of HLA-E and NK cells in the lymph nodes of patients with B cell malignancies. (a) FFPE diagnostic lymph node biopsies from consented patients with indicated B cell malignancies were stained with antibodies for HLA-E or CD20 by immunohistochemistry. Images representative of ≥2 patients for each disease are shown (10× magnification, scale bar 50 μm). The insert in each image represents 1× magnification taken from the same section. Gene expression associated with resting (b) and activated (c) NK cells extracted from Cibersort X quantified as gene set variation analysis score for DLBCL patients in the GOYA trial (*n* = 552) treated with rituximab or obinutuzumab plus CHOP chemotherapy who survived <12 months, 12–36 months, and >36 months. Statistical significance was calculated using Student’s *t*-test in Python. *P < .05 and **P < .01.

To investigate the expression of HLA-E in B cell malignancies, we assessed lymph node FFPE sections from CLL, Burkitt, DLBCL, mantle cell and FL patients by immunohistochemistry. In all patient lymph node samples examined, HLA-E staining was observed on CD20+ cells (Fig. 3a). The specificity of HLA-E staining was confirmed using control sections (Supplementary Fig. S2).

We then analysed data from 552 patients with DLBCL treated with rituximab or obinutuzumab plus chemotherapy from the GOYA trial (NCT01287741). Interestingly, patients who survived >36 months had significantly higher expression of both resting (*P* < .05, Fig. 3b) and activated (*P* < .01, Fig. 3c) NK cell-associated genes (derived from the digital cytometry tool Cibersort X) in the lymphoma microenvironment at pretreatment compared to patients who survived <12 months. This result was also confirmed when applying an alternative NK cell-associated gene list derived from Huntington et al. [3] and Rebuffet et al. [31] (Supplementary Fig. S3; Supplementary Table S4). Together, this data demonstrates that both HLA-E positive tumour cells and NK cells are present in the tumour microenvironment of lymphoma patients.

**Figure 4.**
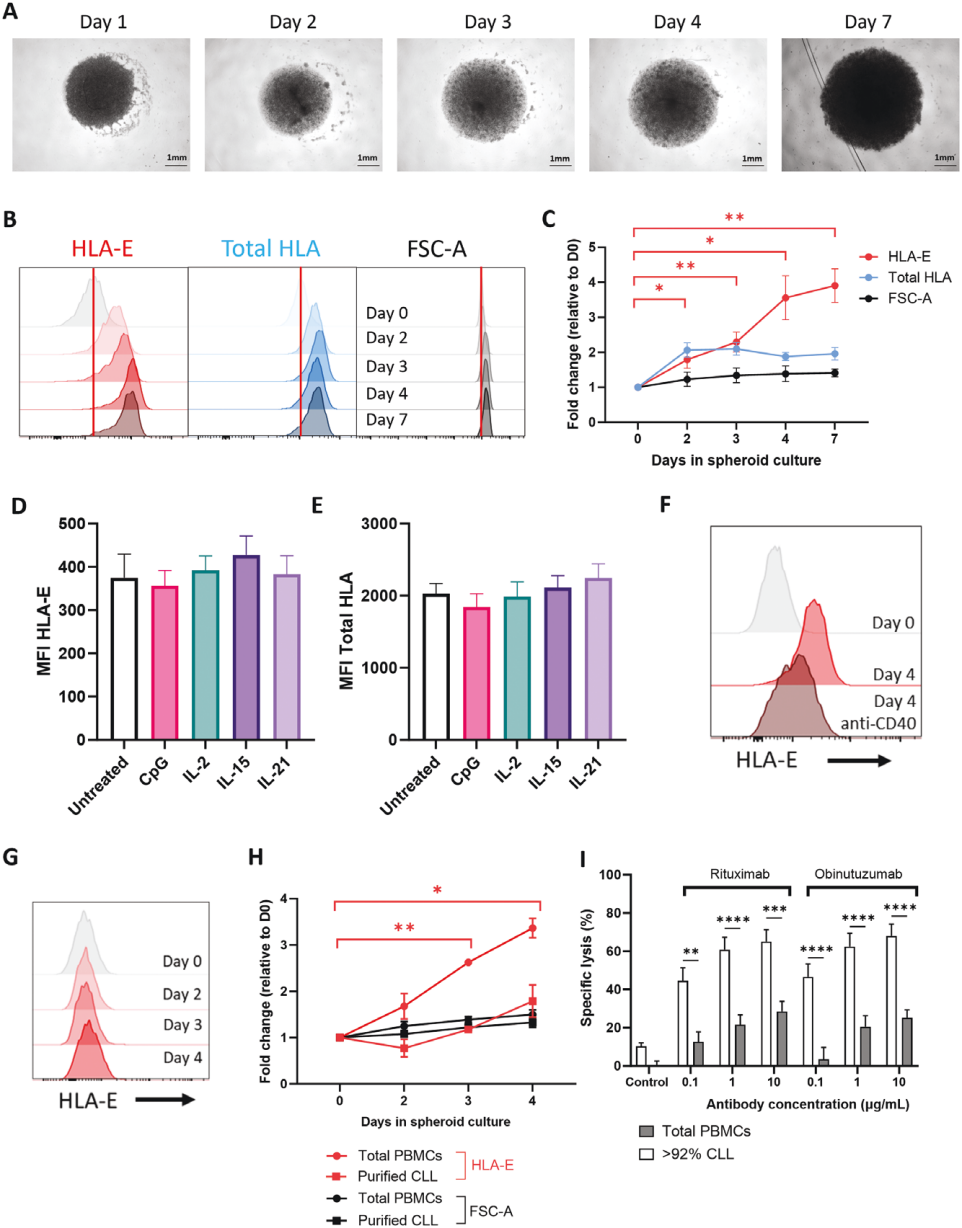
HLA-E expression increases on CLL cells in a patient-derived 3D spheroid model of the lymph node microenvironment. 3D spheroids were generated from CLL patient PBMCs (>10% CD4+ T cells). Spheroids were cultured in ULA plates for up to 7 days with CpG (1 μg/ml), IL-2, IL-15, and IL-21 (all 25 ng/ml). (a) Representative images of spheroids taken at X4 magnification over time (scale bar 1 mm). Expression of HLA-E and total HLA on CLL tumour cells (CD19+CD5+) of the spheroid culture was assessed over time by flow cytometry, alongside FSC-A. Representative FACS plots shown in (b) and data is summarized as fold change expression relative to day 0 as mean±SEM (*n* = 7–9) in (c). (d and e) Primary CLL samples were cultured in 2D with CpG, IL-2, IL-15, and IL-21 individually or left untreated for 24 h before HLA-E (d) and total HLA (e) expression was assessed by flow cytometry. Data are presented as mean±SEM (*n* = 8). (f) Spheroids were cultured in the presence or absence of CD40 antagonist (anti-CD40) for 4 days and HLA-E expression was measured on the CLL cells by flow cytometry. Data is representative of two donors. (g and h) Purified CLL cells (>95% tumour) were cultured as above to generate spheroids in the absence of T cells. HLA-E expression on CLL tumour cells (CD19+CD5+) of the spheroid culture was assessed over time by flow cytometry. Representative FACS plots shown in (g) and data are summarized as mean±SEM in (h) (*n* = 3). (i) Spheroids generated from CLL samples with total PBMCs or >92% tumour were co-cultured with NK cells from healthy donors in combination with rituximab, obinutuzumab, or isotype control for 48 h at a 1:1 E:T ratio. Data are summarized as mean±SEM. Statistical significance was calculated using two-way or one-way ANOVA with Dunnett’s or Sidak’s correction for multiple comparisons. *P < .05, **P < .01, ***P < .001, ****P < .0001.

To investigate the mechanism for HLA-E expression in the lymph node microenvironment in B cell malignancies further, we utilized a CLL patient PBMC-derived spheroid model that partly mimics the lymph node microenvironment and induces CLL proliferation [27]. CLL 3D spheroids were cultured for up to 7 days (Fig. 4a) and ligand expression on the CLL cells was assessed by flow cytometry (Supplementary Fig. S4). HLA-E and total HLA expression increased on the surface of CLL cells (Fig. 4b), and by day 7, HLA-E expression was significantly higher compared to day 0 (*P* < .01) (Fig. 4c). The changes in FSC-A and total HLA were less pronounced, indicating the relative increase in HLA-E expression was not proportional to an increase in cell size or characteristic of all markers. As the generation of the CLL spheroids involved the addition of multiple factors (CpG, IL-2, IL-15, and IL-21), we tested the effect of each factor individually on the expression of HLA-E. Individually, none of these affected HLA-E (Fig. 4d) or total HLA (Fig. 4e) expression on the surface of CLL cells after 24 hours or over longer culture up to 4 days (Supplementary Fig. S5). CD40L and IL-4 are typically T cell-derived in the lymph nodes [19, 32] and T cells in this model were shown to express CD40L (Supplementary Fig. S6). In accordance with this, CD40 antagonism impeded HLA-E upregulation in this model (Fig. 4f). We also tested the contribution of non-CLL cells on HLA-E expression in the 3D spheroid. CLL cells were purified by magnetic-activated cell sorting to remove non-CLL cells (>95% tumour). In these samples, there was no significant change in HLA-E expression in the cells over time relative to day 0 during the 3D spheroid culture (Fig. 4g and h). Furthermore, NK cell-mediated ADCC of spheroids generated from total PBMCs (with a T cell population (Supplementary Fig. S4)) was suppressed relative to spheroids generated from CLL PBMC samples with a negligible T cell population (>92% tumour) (Fig. 4i). This demonstrates that the increase in HLA-E expression on the surface of CLL cells in this CLL lymph node model is reliant on other cells present in the CLL PBMC samples, most likely T cells, which signal to protect these cells from NK cell-mediated ADCC.

### NKG2A blockade overcomes suppression of NK cell-mediated ADCC by CD40L and IL-4 *in vitro*

**Figure 5.**
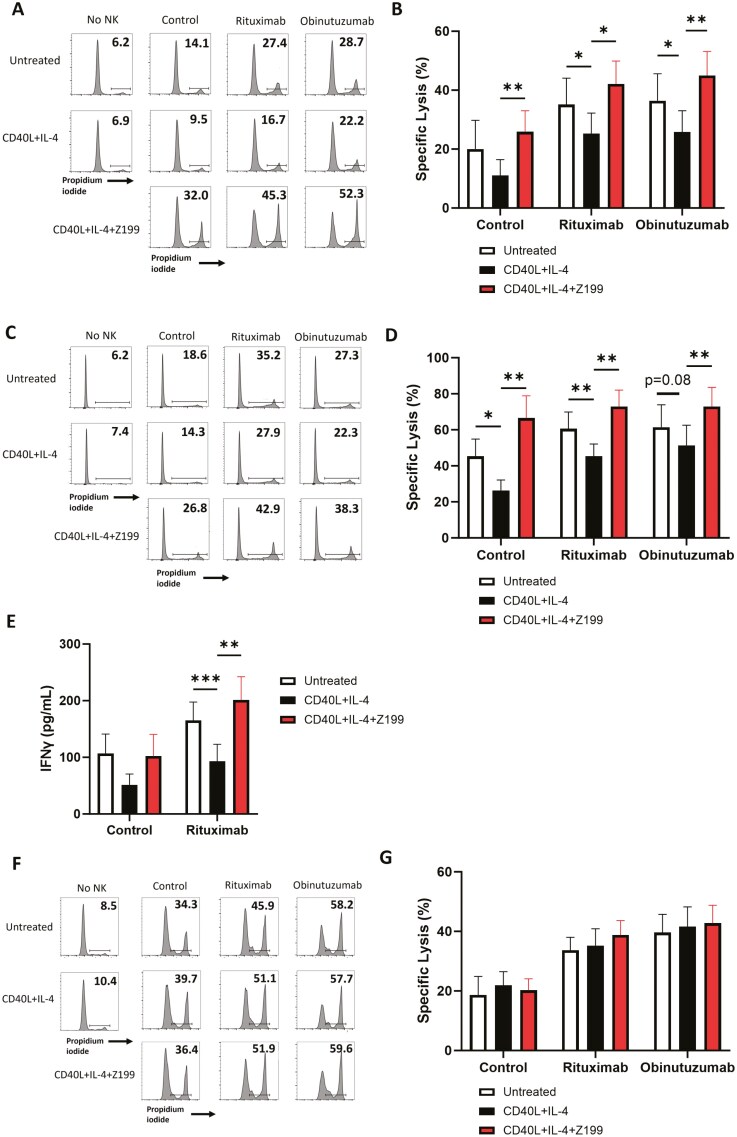
Suppression of NK cell-mediated ADCC by CD40L and IL-4 can be overcome by NKG2A blockade. (a–d) Target cells were treated with 300 ng/ml CD40L and 10 ng/ml IL-4 or left untreated for 24 h before incubation with rituximab (1 μg/ml) or obinutuzumab (0.1 μg/ml) or isotype control (1 μg/ml). NK cells from healthy donors were incubated with 10 μg/ml Z199 (anti-NKG2A) before co-culture with the target cells at a 1:5 (Ramos a and b) or 1:1 (Raji c and d) E:T ratio. Representative FACS plots of raw lysis data are shown in (a) and (c) and NK cell-specific lysis is summarized as mean±SEM in (b) and (d) (*n* = 6–7). (e) Secretion of IFNγ by NK cells co-cultured with Raji as above was assessed by ELISA and data are summarized as mean±SEM (*n* = 10). (f and g) 721.174 cells were treated with 300 ng/ml CD40L and 10 ng/ml IL-4 for 24 h before incubation with rituximab (1 μg/ml) or obinutuzumab (0.1 μg/ml) or isotype control (1 μg/ml). NK cells from healthy donors were incubated with 10 μg/ml Z199 (anti-NKG2A) before co-culture with the target cells at a 1:10 E:T ratio. Representative FACS plots of raw lysis data are shown in (f) and NK cell-specific lysis is summarized as mean±SEM in G (*n* = 5). Statistical significance was calculated using two-way ANOVA with Dunnett’s correction for multiple comparisons. *P < .05, **P < .01, ***P < .001.

As HLA-E is highly expressed in the lymph nodes of patients with B cell malignancies and serves to limit NK cell effector functions, including ADCC, disrupting the HLA-E:NKG2A axis using NKG2A-blocking antibodies could provide a means to increase the efficacy of anti-CD20 mAb therapies. In combination with anti-CD20 mAbs, CD40L, and IL-4 significantly decreased NK cell specific lysis of Ramos (*P* < .05) (Fig. 5a and b) and Raji (*P* < .01 with rituximab) (Fig. 5c and d) cells, while NKG2A-blocking antibody Z199 significantly increased specific lysis (Ramos *P* < .05 with rituximab, *P* < .01 with obinutuzumab and Raji *P* < .01). This effect was also seen against primary CLL in 2D (Supplementary Fig. S7a and b) and 3D (Supplementary Fig. S7c and d) co-culture assays. In addition to NK cell-specific lysis, IFNγ secretion by NK cells was lower in response to CD40L and IL-4-treated targets (*P* < .001) and was increased by Z199 (*P* < .01) in combination with rituximab (Fig. 5e). NKG2A blockade did not alter ADCC against 721.174 cells in combination with rituximab or obinutuzumab (Fig. 5f and g), supporting that the effect of NKG2A blockade is specific to targets expressing surface HLA-E. To further assess the importance of the HLA-E:NKG2A immune checkpoint in this setting, we used the exportin-1 inhibitor selinexor, which has previously been shown to selectively downregulate surface expression of HLA-E on malignant B cells in the presence of CD40L and IL-4 (23). Selinexor enhanced NK cell-specific lysis (*P* < .01) and rituximab-mediated ADCC (*P* < .01) of Raji cells in the presence of CD40L and IL-4 (Supplementary Fig. S8a and b).

**Figure 6.**
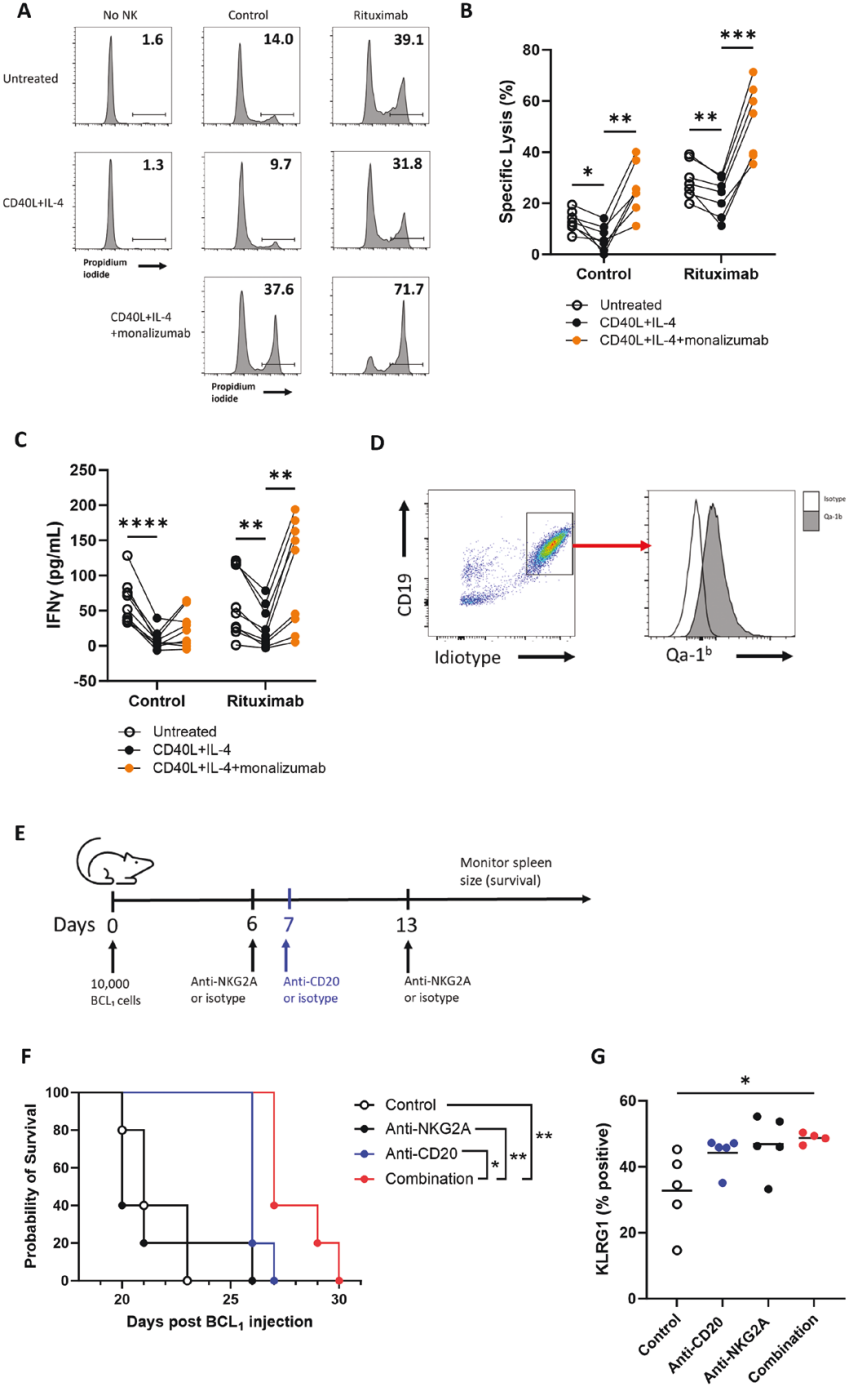
Clinically relevant anti-NKG2A monalizumab enhances NK cell-mediated ADCC *in vitro* and NKG2A blockade improves anti-CD20 mAb therapy *in vivo*. Raji cells were treated with 300 ng/ml CD40L and 10 ng/ml IL-4 or left untreated for 24 h before incubation with rituximab or isotype control (1 μg/ml). NK cells from healthy donors were incubated with 15 μg/ml monalizumab (anti-NKG2A) before co-culture with the treated Raji cells at a 1:1 E:T ratio. Representative FACS plots of raw lysis data are shown in (a) and NK cell-specific lysis is summarized in (b) (*n* = 6). (c) Secretion of IFNγ by NK cells co-cultured with Raji as above was assessed by ELISA (*n* = 9). Statistical significance was calculated using two-way ANOVA with Dunnett’s correction for multiple comparisons. (d) Expression of Qa-1^b^ on BCL₁ murine lymphoma cells (CD19+Idiotype+) was confirmed by flow cytometry. Open histogram indicates isotype control-stained cells and the shaded histogram indicates Qa-1^b^ stained cells. (e) 10^4^ BCL₁ cells were injected into mice i.v. before injection with anti-NKG2A on days 6 and 13 (200 μg, i.v.) and anti-CD20 on day 7 (200 μg, i.p.) or respective isotype controls. Tumour growth was monitored by palpation of spleens and mice were terminated once humane endpoint was reached. (f) Kaplan–Meier survival curve of BCL₁-bearing mice treated with isotype controls (control), anti-CD20, anti-NKG2A, or anti-CD20 and anti-NKG2A antibodies (combination). Statistical significance calculated with the log-rank (Mantel-Cox) test, *n* = 5 per group. (g) Blood was withdrawn from mice via tail lancing two days after the last injection of anti-NKG2A (day 15), and KLRG1 expression was assessed on murine NK cells (CD3-NKp46+CD49b+) by flow cytometry. Statistical significance calculated with one-way ANOVA with Tukey’s correction for multiple comparisons. *P < .05, **P < .01, ***P < .001, ****P < .0001.

The anti-NKG2A antibody monalizumab is in phase III clinical trials in combination with tumour-targeting antibodies for patients with nonsmall cell lung cancer (NCT05221840). Consistent with experiments using Z199, monalizumab significantly increased specific lysis (Fig. 6a and b) and IFNγ secretion (Fig. 6c) against NHL cells treated with CD40L and IL-4 in both the presence and absence of rituximab. Overall, these data demonstrate that disruption of the HLA-E:NKG2A axis via NKG2A blocking antibodies, including those under clinical investigation, increases NK cell-mediated ADCC against malignant B cells in the presence of CD40L and IL-4.

### Anti-NKG2A improves anti-CD20 therapy *in vivo*

We next tested as proof-of-concept whether NKG2A blockade could improve efficacy of anti-CD20 therapy *in vivo*. BCL_1_ murine B cell lymphoma cells express Qa-1^b^ (Fig. 6d), the ligand for NKG2A in mice [33], thus we tested the effect of NKG2A blockade in this model. Mice were injected with 10^4^ BCL_1_ cells i.v. followed by antimouse NKG2A or isotype control i.v. on days 6 and 13, and antimouse CD20 i.p. or isotype control on day 7 post-tumour injection (Fig. 6e). There were no statistically significant differences in the percentage of NK cells in the peripheral blood (Supplementary Fig. S9a) or spleen (Supplementary Fig. S9b) between mice of different treatment groups. Anti-NKG2A alone did not improve survival relative to the control group, in line with previous reports [24]. Anti-CD20 extended survival and combining anti-NKG2A with anti-CD20 resulted in a statistically significant increase in survival compared to the control (*P* < .01), anti-NKG2A alone (*P* < .01) and anti-CD20 alone (*P* < .05) (Fig. 6f). In addition, only mice that received the combination of anti-CD20 and anti-NKG2A had statistically significant evidence of NK cell activation as determined by KLRG1 expression (*P* < .05) (Fig. 6g). Overall, these data indicate that NKG2A blockade can improve the efficacy of anti-CD20 and activation of NK cells *in vivo.*

## Discussion

Anti-CD20 antibodies are widely used as first-line treatments for patients with B cell malignancies and are known to elicit cytotoxicity via NK cells in humans. The lymph nodes represent a pro-tumour niche and a source of minimal residual disease in these pathologies, however most studies assessing the NK cell-mediated function of anti-CD20 antibodies have used conditions which do not account for the lymph node microenvironment. Our study shows the lymph node-associated molecules CD40L and IL-4 inhibit NK cell cytotoxicity of malignant B cells in combination with anti-CD20 therapy via the HLA-E:NKG2A checkpoint axis. HLA-E was found to be highly expressed on CD20+ cells in the lymph nodes of patients with a wide range of B cell malignancies and antibody blockade of NKG2A potentiated NK cell function against malignant B cells even in the presence of CD40L and IL-4. This is in accordance with the recognized inhibitory effect of NKG2A on NK cell activation via recruitment of the tyrosine phosphatase SHP-1 (24). As a proof-of-concept, NKG2A blockade *in vivo* increased survival of anti-CD20 mAb-treated mice bearing murine B cell lymphoma. The results of our study indicate that combining NKG2A blockade with anti-CD20 mAb therapy may enhance NK cell-mediated tumour depletion within the lymph node microenvironment and therefore clearance of minimal residual disease.

CD40L and IL-4 are typically derived from T cells within the lymph node microenvironment [19, 32]. Our study revealed that lymph node-mimicking CLL spheroids without T cells lacked the increase in HLA-E expression on the surface of tumour cells compared to T cell-containing CLL spheroids, suggesting that the presence of T cells impairs NK cell-mediated ADCC within the lymph node microenvironment via the HLA-E:NKG2A axis. However, we cannot rule out the contribution of other PBMCs such as monocytes to changes in HLA-E expression and ADCC which were also depleted during CLL purification [27]. The presence of T cells has recently been shown to support NK cell viability, proliferation, and rituximab-mediated ADCC *in vitro* and *in vivo* using a humanized mouse model [34, 35]. These studies indicated that the positive effects observed were likely due to IL-2 secreted by the T cells which is known to activate NK cells and our study therefore adds a further layer of complexity to T cell:NK cell interactions in the lymph node. These previous studies looked at the direct impact of T cells on NK cell function, whereas our study assesses the function of NK cells as a subsequent consequence of T cell interaction with malignant B cells. Together, these data indicate that T cell-derived signalling molecules can both directly support NK cells (through IL-2) and suppress NK cell-mediated ADCC via upregulation of HLA-E on malignant B cells (through CD40L and IL-4).

NK cells represent a relatively small percentage of immune cells within the lymph nodes of patients with B cell malignancies [36], however, previous reports have associated higher expression of NK cell-related genes within the lymphoma microenvironment with progression-free survival in patients treated with obinutuzumab [5]. The current study builds on this by demonstrating an association between both resting and activated NK cell-related gene expression and progression-free survival in lymphoma patients treated with rituximab or obinutuzumab. In addition, rituximab treatment increases intranodal NK cell proliferation and markers of cytotoxicity in FL patients [37], indicating that NK cells in the lymph nodes of lymphoma patients can respond to rituximab. Approximately 50% of intranodal NK cells were NKG2A+, indicating the potential utility of NKG2A blocking antibodies within the lymph node microenvironment. Although other checkpoint inhibitors such as nivolumab (anti-PD-1) and ipilimumab (anti-CTLA-4) have shown remarkable success in certain solid tumours [38], these have not been adopted for treatment of NHLs other than pembrolizumab for patients with relapsed/ refractory primary mediastinal large B cell lymphoma (PMBCL) [39], likely due to lack of efficacy. In addition, NK cell adoptive transfer therapies, including chimeric antigen receptor (CAR)-NK cells, are now under clinical evaluation in patients with B cell malignancies and have shown an overall response rate of 48.6% and an excellent safety profile in recent trials [40]. Adoptive transfer strategies rely on expansion of NK cells *ex vivo* to generate enough cells for therapy [41]. NKG2A expression on the surface of NK cells has been shown to increase with multiple methods of *ex vivo* expansion [42], thus interruption of the HLA-E:NKG2A immune checkpoint axis may be even more relevant in the context of adoptive transfer. In patients, anti-CD19 CAR-NK cells have been reported to preferentially home to the lymph nodes over blood or bone marrow [43] and early efforts to improve lymph node homing of *ex vivo* expanded NK cells by transfection with genes for lymph node-homing receptors are already underway [44]. Furthermore, high HLA-E expression in the lymph nodes of patients with a wide range of B cell malignancies (CLL and mantle cell, Burkitt, DLBCL, and follicular NHLs) indicates disruption of this immune checkpoint may be broadly applicable to a range of NK cell therapies in patients with different types of B cell malignancies.

The NKG2A blocking antibody monalizumab is currently in a phase III clinical trial in combination with durvalumab (anti-PD-L1) for the treatment of patients with nonsmall-cell lung cancer (NCT05221840). Early clinical data have showed excellent safety and promising efficacy in solid tumours [45] and monalizumab has also been evaluated as safe in patients with haematological malignancies when administered after allogeneic haematopoietic stem cell transplantation [46] (NCT02921685). Other NKG2A blocking antibodies are also in development (NCT06094296). Alternatively, CRISPR-Cas9 knock-out of NKG2A in NK cells has been demonstrated to improve anticancer activity of anti-CD33-CAR-NK cells against AML both *in vitro* and *in vivo* [47] and this could be applied to improve CAR-NK cell function within the lymph node microenvironment of B cell malignancies. Targeting HLA-E to improve NK cell cytotoxicity is also under investigation, including HLA-E blocking antibodies [48] and the approved drugs selinexor and bortezomib which can downregulate surface HLA-E [23, 28, 49]. Collectively, these studies reveal a variety of strategies to interrupt the HLA-E:NKG2A axis which may improve the function of NKG2A+ NK cells against malignant B cells within the lymph node microenvironment.

Limitations of this study include species-specific differences in NK cell function between humans and mice [50] and a lack of an HLA-E knock-out experiment to confirm the relevance of HLA-E in the presence of CD40L and IL-4. In addition, although we utilized a primary CLL 3D spheroid model, this does not fully recapitulate the human lymph node microenvironment in CLL or other B cell malignancies [27].

In conclusion, this study identifies that the lymph node-associated signalling molecules CD40L and IL-4 inhibit NK cell-mediated ADCC against malignant B cells and that this can be overcome by disruption of the HLA-E:NKG2A axis. Addition of NKG2A blocking antibodies to anti-CD20 antibody treatment regimens may therefore aid the clearance of tumour cells from within the lymph node niche in patients with B cell malignancies.

## Supplementary Material

ltaf029_suppl_Supplementary_Materials_1

## Data Availability

All data generated or analysed during this study are included in this published article [and its Supplementary Information files] and the raw data will be made freely available upon request.
