# Supplementary material for: CD40L and IL-4 suppress NK cell-mediated antibody-dependent cellular cytotoxicity through the HLA-E:NKG2A axis
Source: Immunother Adv. 2025 Aug 27;5(1):ltaf029. doi: 10.1093/immadv/ltaf029 (PMC12448733; doi:10.1093/immadv/ltaf029)
Supplement: ltaf029_suppl_Supplementary_Materials_1 [file ltaf029_suppl_supplementary_materials_1.pdf]

Supplementary information

Bioinformatics analysis methods

Preliminary bulk RNASeq and gene expression analysis

To examine the significance of NK cells in DLBCL, we analyzed bulk RNASeq data from the GOYA clinical trial (1), comparing rituximab vs obinutuzumab. Data from 553 samples (GEO #GSE125966) included a gene count matrix and metadata, analyzed with R (v4.4.2) and Python (v3.11.11).

Count matrix was normalised using the edgeR R package (version 4.4.2) using trimmed mean of M-values (TMM). Outliers were detected through plotting the interquartile range (IQR) against the median values. For gene set variation and CIBERSORT analyses, genes without matching Entrez IDs were excluded. The metadata used included a variable categorizing progression-free survival (pfstt) into <12, 12–36, and >36 months. Individual gene expression was visualized (after adding a 0.01 pseudo-count for log transformation) as part of the preliminary analysis on the remaining 552 samples.

Gene set variation analysis (GSVA)

The GSVA R package (version 2.0.5) was used for the analysis of the impact of NK cell markers on patient survival. A set of 77 NK-associated genes was compiled for analysis from two studies—Rebuffet et al. (2024) and Huntington et al. (2020) —and analyzed using GSVA with the gene count matrix. To confirm these findings, resting (n = 112) and activated (n = 93) NK signatures from the LM22 immune cell signature matrix (CIBERSORTx, https://cibersortx.stanford.edu/;(2)) were used. We set an NK cell expression threshold >500 to build each NK-associated gene list. Three independent sets of randomly generated genes were analysed alongside all lists of genes of interest, to confirm the validity of the result significance.

The statistical analysis was performed using the statsmodels.stats.multitest (statsmodels version 0.14.4) and scipy.stats (scipy version 1.13.1) Python packages to assess the significance of NK gene expression with a Student's t-test across different progression-free survival periods. T-scores were calculated to signify the magnitudes of differences between survival groups, and p-values were used for a final assessment of significance. The Benjamini-Hochberg procedure was used for p-value correction for multiple hypothesis testing.

References

1. Sehn LH, Martelli M, Trněný M, Liu W, Bolen CR, Knapp A, et al. A randomized, open-label, Phase III study of obinutuzumab or rituximab plus CHOP in patients with previously untreated diffuse large B-Cell lymphoma: final analysis of GOYA. J Hematol Oncol [Internet]. 2020 Jun 6 [cited 2024 Oct 31];13(1):71. Available from: https://pmc.ncbi.nlm.nih.gov/articles/PMC7276080/

2. Newman AM, Steen CB, Liu CL, Gentles AJ, Chaudhuri AA, Scherer F, et al. Determining cell-type abundance and expression from bulk tissues with digital cytometry. Nat Biotechnol [Internet]. 2019 Jul 1 [cited 2025 Feb 5];37(7):773. Available from: https://pmc.ncbi.nlm.nih.gov/articles/PMC6610714/

Supplementary figures

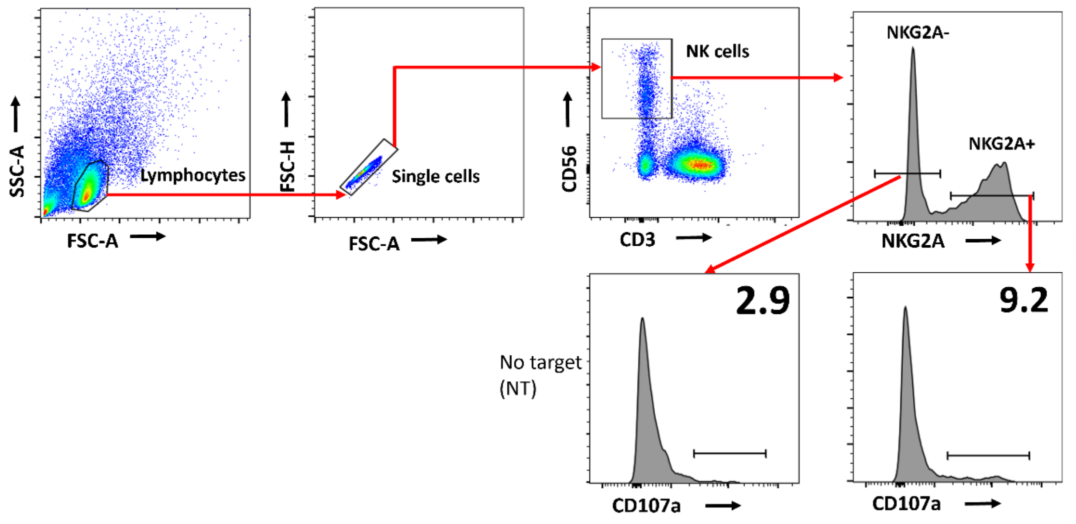

Figure S1: Gating strategy to assess degranulation of NKG2A+ and NKG2A- NK cells by flow cytometry.
NK cells (CD56+CD3-) were identified from the lymphocyte and single cell gate. NKG2A+ and NKG2A- NK cells were then gated. The no target (NT) controls in the degranulation assays were used to set the gates for CD107a expression of the NKG2A+ and NKG2A- NK cell populations.

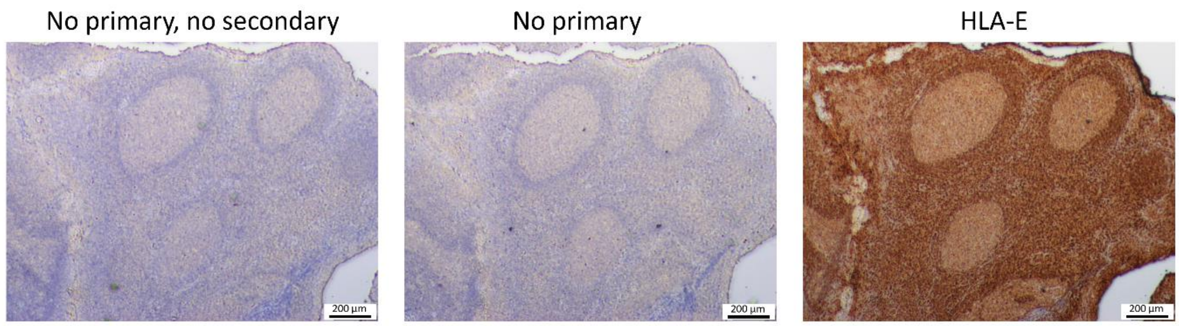

Figure S2: Immunohistochemistry HLA-E staining controls.
FFPE tonsil sections from a consented patient were stained with no primary or secondary antibody, no primary anti-HLA-E antibody or with HLA-E antibody with secondary antibody by immunohistochemistry. Images taken at 4x magnification using an Olympus CKX41 microscope.

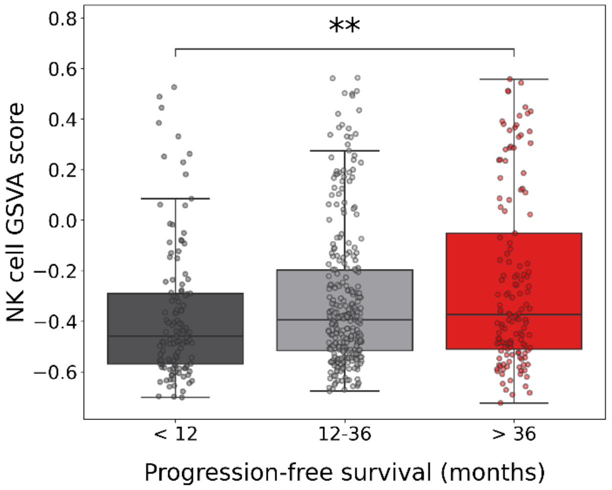

Figure S3: Higher expression of NK cell-associated genes in DLBCL patients with >36 months progression-free survival in the GOYA trial.
NK cell-associated gene expression quantified as gene set enrichment (GSVA) score for DLBCL patients in the GOYA trial treated with rituximab or obinutuzumab plus CHOP chemotherapy. The gene signature for the analysis was derived from NK-associated gene lists by Rebuffet et al. (2024) and Huntington et al. (2020). Statistical significance was calculated using students t-test **p<0.01.

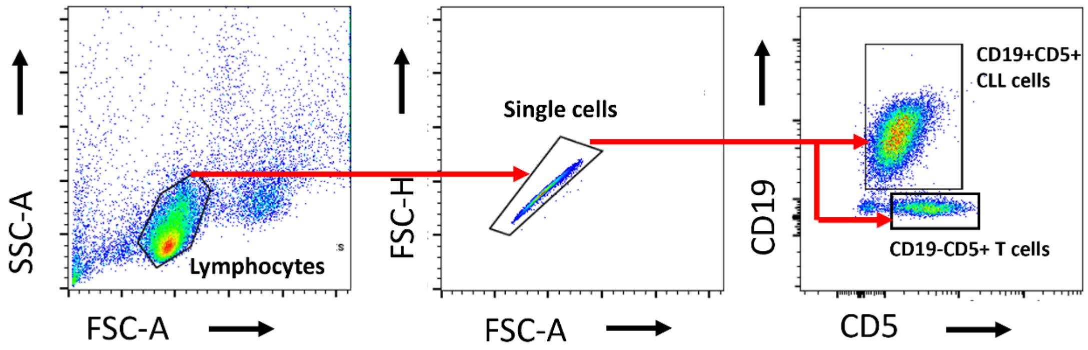

Figure S4: Gating strategy to identify tumour cells in PBMC samples from CLL patients by flow cytometry.
CLL tumour cells (CD19+CD5+) were identified from the lymphocyte and single cell gate. CD19-CD5+ cells were identified as T cells.

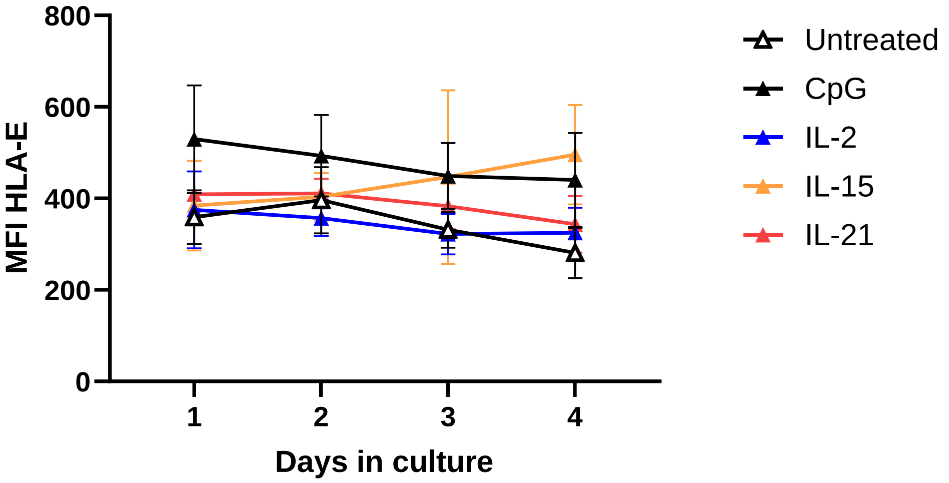

Figure S5: HLA-E expression on CLL cells treated with CpG, IL-2, IL-15 or IL-21 individually.
Primary CLL samples were cultured in 2D with CpG (1 µg/mL), IL-2, IL-15 and IL-21 (all 25 ng/mL) individually or left untreated for up to 4 days. HLA-E expression on the CLL cells (CD19+CD5+) was measured by flow cytometry (n=3). Statistical significance was calculated using two-way ANOVA using Dunnet's correction for multiple comparisons.

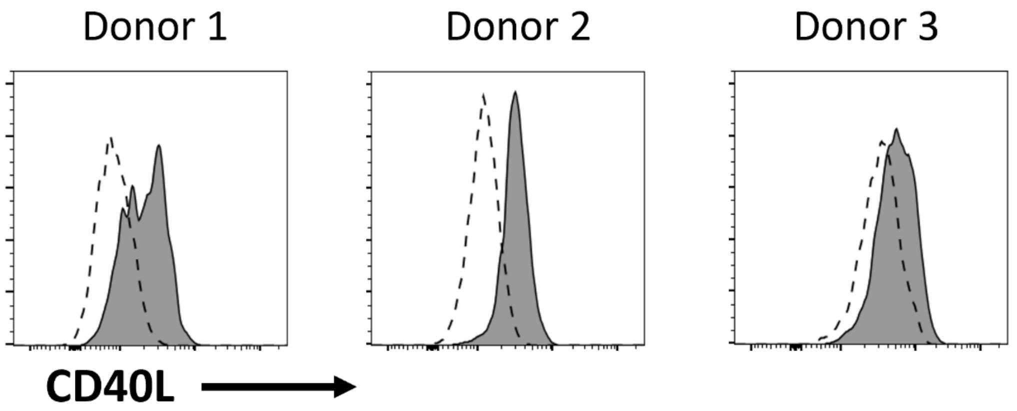

Figure S6: CD40L staining of T cells within the 3D CLL spheroid model.
3D spheroids were generated from CLL patient PBMCs from 3 donors (>10% CD4+ T cells). Spheroids were cultured in ULA plates for up to 7 days with CpG (1 µg/mL), IL-2, IL-15 and IL-21 (all 25 ng/mL). CD40L expression was measured on the T cells (CD5+CD19-) by flow cytometry on day 3 after initial spheroid formation. Dotted histograms represent isotype control stain and shaded histograms represent CD40L stain.

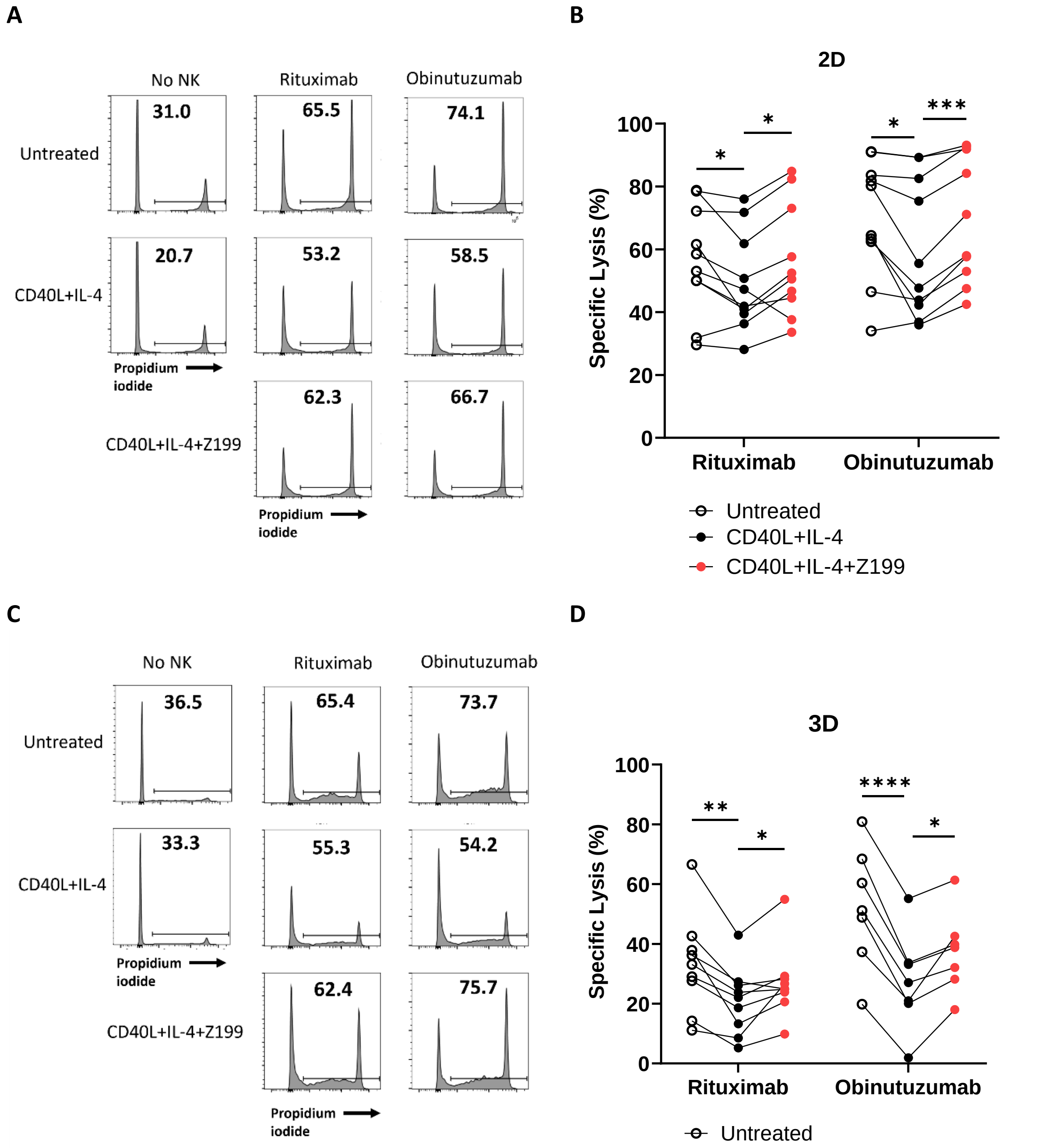

Figure S7: Suppression of NK cell-mediated ADCC of primary CLL cells by CD40L and IL-4 can be overcome by NKG2A blockade.

Primary CLL cells were treated with 300 ng/mL CD40L and 10 ng/mL IL-4 for 24 hours before incubation with rituximab (10 µg/mL) or obinutuzumab (1 µg/mL) in either (A, B) conventional 96 well plates (2D) or (C, D) ULA plates (3D). NK cells from healthy donors were incubated with 10 µg/mL Z199 (anti-NKG2A) before co-culture with the CLL cells at a 1:1 E:T ratio. Representative FACS plots of raw lysis data shown in A and C and NK cell specific lysis is summarised in B and D (n=9). Statistical significance was calculated using two-way ANOVA with Dunnett's correction for multiple comparisons. *P<0.05, **p<0.01, ***p<0.001, ****p<0.0001.

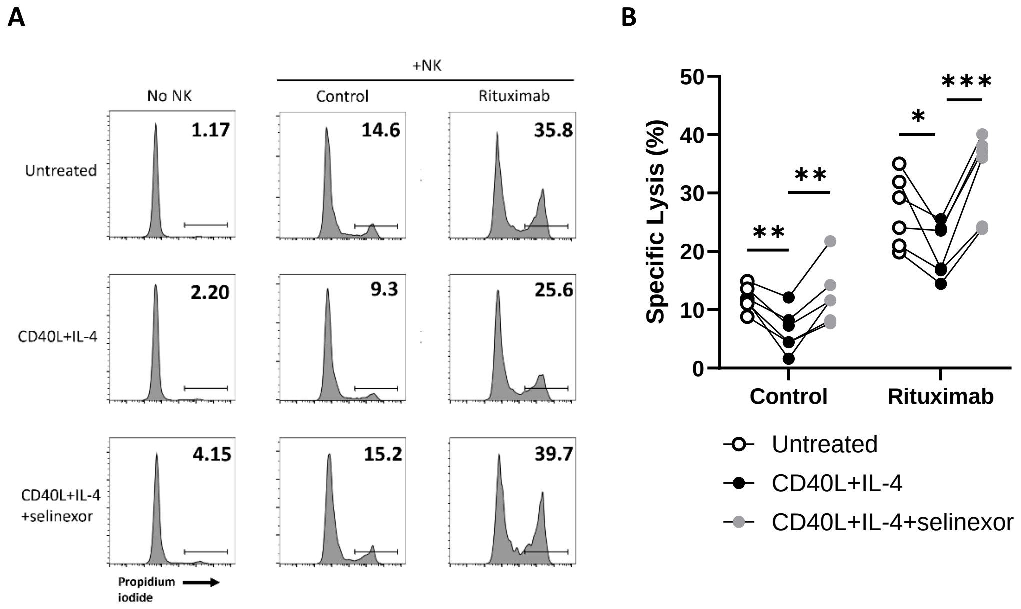

Figure S8: Selinexor enhances NK cell-mediated ADCC of Raji cells in the presence of CD40L and IL-4.
Raji cells were treated with 300 ng/mL CD40L and 10 ng/mL IL-4 for 30 min before incubation with 500 nM selinexor or DMSO control. After 24 hours, the Raji cells were incubated with 1 µg/mL rituximab before NK cells from healthy donors were added at a 1:1 E:T ratio. Representative data shown in A and summarised data shown in B (n=6).

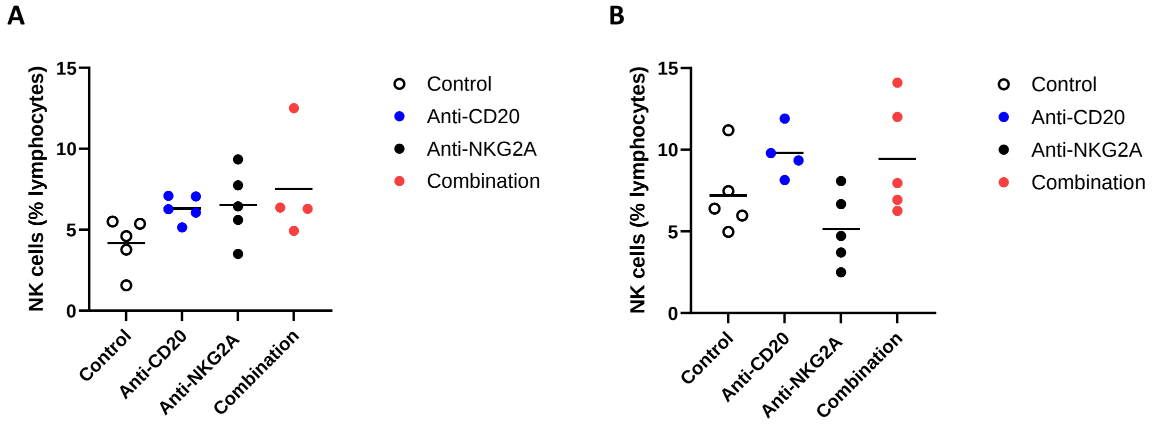

Figure S9: Percentage of NK cells in BCL_1-bearing mice receiving mAb therapy.
10^4 BCL_1 cells were injected into mice i.v. before injection with anti-NKG2A on days 6 and 13 (200 µg, i.v.) and anti-CD20 on day 7 (200 µg, i.p.) or respective isotype controls. Percentage of NK cells in (A) peripheral blood at day 15 post tumour injection and (B) spleen at experimental endpoint for each mouse was measured by flow cytometry (n=4-5). Statistical significance calculated with one-way ANOVA with Tukey's correction for multiple comparisons.

Supplementary tables

Supplementary Table 1: CLL patient information for PBMC samples.
IGHV = Immunoglobulin heavy chain variable region. M = Mutated IGHV. U = Unmutated IGHV. N/A = not available.

Patient number	Binet Stage	Rai stage	IGHV	Tumour in peripheral blood (%)
**1451**	A	1	M	94.0
**1348**	A	N/A	M	92.1
**1514**	N/A	N/A	U	72.0
**584C**	A	0	M	90.5
**1471**	A	0	U	92.0
**1423**	B	1	U	96.8
**641E**	A	1	M	67.0
**709A**	A	0	M	76.4
**1049A**	A	0	M	74.0
**1312**	N/A	N/A	M	70.7
**654C**	A	0	M	66.0
**572A**	A	0	M	50.6
**832A**	A	0	M	56.0
**885**	N/A	0	M	54.0
**1055**	N/A	0	M	96.0
**1187**	N/A	0	M	92.2
**1209**	N/A	0	M	76.9
**1336**	N/A	1	M	69.5

Table S2: Fluorescently conjugated antibodies for flow cytometry assays.				
**Surface marker**	**Fluorochrome**	**Clone**	**Supplier**	**Concentration (μg/mL)**
Primary CLL and cell lines				
**HLA-E**	PECy7	3D12	Biolegend	6.0
**HLA-A/B/C (total HLA)**	AF488	W6/32	Biolegend	0.3
**CD19**	Pacific blue/PE	HIB19	Biolegend	2.0
**CD5**	PerCP	UCHT2	Biolegend	1.0
**CD40L**	APC	24-31	Biolegend	5.0
Healthy donor PBMCs				
**CD3**	PerCP	UCHT1	Biolegend	1.0
**CD56**	PE/Cy7	HCD56	Biolegend	4.0
**NKG2A**	FITC/ Vio Bright V423	REA110	Miltenyi Biotech	1.0
Murine cells				
**CD19**	PerCPcy5.5	6D5	Biolegend	2.0
**BCL-1 Idiotype**	Alexa Fluor 488	6A5	In house	10.0
**Qa-1^{b}**	PE	6A8.6F10.1A6	BD Pharmigen	8.0
**CD3**	PE	145-2C11	Biolegend	2.0
**NKp46**	Brilliant Violet 421	29A1.4	Biolegend	8.0
**CD49b**	FITC	DX5	Biolegend	8.0
**KLRG1**	PE/Cy7	2F1/KLRG1	Biolegend	2.0
**Isotype**	PE	MOPC-21	Biolegend	8.0

Table S3: Patient information for lymph node samples.		
**Disease**	**Type of lymph node**	**Diagnosis/disease stage**
**CLL**	Neck node	In blood/marrow
**Burkitt**	Groin node	Unknown stage
**DLBCL**	Supra clavicular node	Unknown stage
**Mantle cell**	Groin node	Stage 3a
**Follicular**	Submandibular node	Unknown stage

Table S4: List of NK cell-associated genes extracted from Huntington *et al.*, 2020 and Rebuffet *et al.*, 2024 and used in GSVA analysis of samples (n=552) from the GOYA DLBCL clinical trial.

Cytokines and chemokines	Chemotaxis receptors	Activating receptors	Inhibitory receptors	Cytokine receptors	Additional receptors	Cytotoxic molecules	Cell maturation
IFNG	S1PR1	KLRK1	KLRC1	TGFBR1	NT5E	FASLG	ITGAM
CCL4	CXCR3	KLRC2	CD300A	IL12RB1	ITGAL	GSDMD	ITGAX
CCL4L2	CXCR4	NCR1	TIGIT	IL2RG	KLRC4	GZMB	NCAM1
IL32	S1PR5	CD160	KIR3DL2	TGFBR3	KLRG2	NKG7	FCGR3A
IL16	CX3CR1	NCR3	KIR3DL1	TGFBR2	TNFRSF18	PRF1	ITGB2
CCL3	CXCR2	SLAMF6	KIR2DL3	IL10RA		GZMA	
CCL5	S1PR4	SLAMF7	KIR2DL1	IL18R1		GZMH	
FLT3LG		CD226	KIR3DL3	IL18RAP		GZMK	
XCL1		CD244	KLRB1	IL2RB		TNFSF10	
XCL2		TMIGD2	SIGLEC7	IL10RB			
		NCR2	HAVCR2				
		KIR2DS4	ADORA2A				
		KIR2DL4	PDCD1				
		KLRC3	KLRD1				
		KLRF1	KLRG1				
		CD69					
